# Randomized controlled trial comparing telephone and mail follow-up for recruitment of participants into a clinical trial of colorectal cancer screening

**DOI:** 10.1186/1745-6215-14-40

**Published:** 2013-02-11

**Authors:** Arthur D Wong, John Kirby, Gordon H Guyatt, Paul Moayyedi, Parag Vora, John J You

**Affiliations:** 1 Department of Medicine, McMaster University, Hamilton, ON, Canada; 2 Department of Radiology, McMaster University, Hamilton, ON, Canada; 3 Department of Clinical Epidemiology & Biostatistics, McMaster University, Hamilton, ON, Canada

**Keywords:** Randomized controlled trial, Recruitment, Telephone, Mail, Colorectal cancer screening

## Abstract

**Background:**

Investigators often face challenges when recruiting participants into randomized controlled trials (RCTs). Some data suggest that telephone reminders may lead to greater participant enrollment.

**Methods:**

Patients aged 50 to 70 years from family practice rosters were initially mailed invitations to participate in an RCT of colorectal cancer screening. Patients who did not respond were randomly allocated to follow-up invitations by either telephone or mail four weeks after the initial invitation. The primary outcome was attendance for eligibility screening with the study nurse.

**Results:**

After mailing invitations to 1,348 patients, 104 patients were initially enrolled in the RCT of colon cancer screening. Of 952 patients who did not respond to the initial mailed invitation, we randomly allocated 480 to follow-up invitation by telephone and 472 to follow-up invitation by mail. Attendance for eligibility screening with the study nurse was more frequent when non-responders were followed-up by telephone (84/480, 17.5%) than by mail (43/472, 9.1%) (relative risk (RR) 1.92, 95% confidence interval (CI) 1.36 to 2.71, *P* < 0.001). Enrollment into the RCT was also greater among patients followed-up by telephone (59/480, 12.3%) compared to those followed-up by mail (35/472, 7.4%) (RR 1.66, 95% CI 1.11 to 2.47, *P*=0.01).

**Conclusions:**

Telephone-based follow-up results in greater enrollment compared to a mail-based method. Our findings should be of interest to investigators conducting RCTs, particularly trials of screening interventions involving asymptomatic participants for which volunteer participation may be challenging.

**Trial registration:**

Clinicaltrials.gov NCT00865527

## Background

Investigators often face challenges when recruiting participants into randomized controlled trials (RCTs). Inadequate recruitment may prevent studies from detecting significant intervention effects [1], cause delays, increase costs, and result in failure to complete trials [2-4]. An understanding of effective strategies to improve recruitment into clinical trials is particularly relevant for trials of screening interventions, in which participants do not have overt, symptomatic disease so may not be as eager to participate in clinical studies.

A recent Cochrane review concluded that telephone reminders to non-responders, the use of opt-out rather than opt-in procedures (patients have to contact their physician to withhold contact details), and open designs (participants are informed about the treatment they are receiving in the trial) were all effective strategies in improving recruitment of participants to randomized clinical trials. However, some of these strategies have disadvantages. Opt-out procedures are controversial and open designs are unblinded [5].

Some data suggest that telephone follow-up may lead to greater participant enrollment. The Prostate, Lung, Colorectal and Ovarian (PLCO) cancer screening trial demonstrated that the two centers with the highest yield incorporated telephone-based strategies in their recruitment methods: Salt Lake City investigators recruited other household members during follow-up telephone interviews, while Minnesota investigator increased their enrollment rates by 15% through follow-up telephone calls to non-responders four weeks after their initial mail invitations [6].

Furthermore, two RCTs have examined the effectiveness of telephone reminders on recruitment. One study compared telephone reminders to no reminders on recruiting potential participants who did not respond to an initial mail invitation [7]. Another study compared telephone reminders to mail reminders on recruiting patients to an observational study [8]. The two RCTs found that telephone reminders can increase recruitment by nearly two-fold [5,7,8]. Although promising, telephone-based follow-up is likely to be more time consuming and more costly than alternatives such as mail-based follow-up. Given that only one RCT has directly compared the effectiveness of telephone to mail reminders on recruitment, and that these investigators examined recruitment into an observational study and not into an interventional trial [8], the extent to which telephone-based follow-up can increase enrollment compared to mail-based follow-up, particularly into an RCT for screening asymptomatic individuals, remains unclear.

In this paper, we compare the effectiveness of telephone versus mail-based follow-up using recruitment data from the SCOPE trial (Screening for Colorectal Cancer: a randomized trial of virtual colonoscopy, optical colonoscopy and fecal occult blood testing). The SCOPE trial is a study assessing the feasibility of conducting an RCT comparing three colorectal cancer screening interventions among participants enrolled from primary care practices in Ontario, Canada. To recruit patients into the SCOPE trial, potentially eligible patients (that is aged 50 to 70 years old) received a personalized, mailed invitation signed by their primary care physician to take part in colorectal cancer screening. This was based on successful recruitment strategies used in earlier cancer screening studies in our jurisdiction [9-11]. For individuals who did not respond to this mailed invitation, we were uncertain whether telephone or mail follow-up would result in greater enrollment rates.

Therefore, the objective of the current study was to directly compare, among individuals who did not respond to an initial mailed invitation to take part in the SCOPE trial, the effects of telephone versus mail follow-up at four weeks on attendance rates with the study nurse for eligibility screening and enrollment rates into the SCOPE trial. We hypothesized that patients randomized to telephone-based follow-up would be more likely to attend for eligibility screening and be enrolled into the SCOPE trial than those randomized to mail-based follow-up.

## Methods

### Design

A parallel group, randomized controlled trial.

### Study population

Using a recruitment approach modeled after the successful strategies used in earlier cancer screening studies in our jurisdiction [9-11], patients aged 50 to 70 years from five participating family practice rosters were mailed invitations, printed on their family physician’s letterhead and signed by their family physician, to participate in a study of colorectal cancer screening (the SCOPE trial). Non-responders at four weeks after the initial mailing were eligible for the current recruitment substudy. To avoid contamination, if more than one non-responder lived at the same address (for example, a married couple), we randomly selected only one individual from a given address for inclusion in this substudy. Individuals were excluded from the current study if their initial invitation was ‘returned to sender’ due to an invalid mailing address.

### Randomization procedure

A de-identified list of eligible participants (that is a list of unique study numbers corresponding to the eligible participants but containing no personal identifiers) was prepared by the study nurse. To ensure concealment of allocation, one of the investigators (JJY), who was not directly involved in the recruitment of the patients from family physicians’ offices and who was unaware of the patients’ identities, produced a computer generated randomization sequence (block size 4) stratified by family practice. This investigator (J.J.Y.) then allocated each unique study number to either telephone or mail follow-up (in a 1:1 ratio) according to the randomization sequence and returned the participant allocation list to the study nurse.

### Study interventions

Patients randomized to telephone follow-up received up to three telephone calls from the same study nurse, each on separate days at different times of the day (morning, mid-day and afternoon) over a period of one to two weeks, beginning at four weeks after the initial invitation. If a third telephone call was not picked up, the study nurse left a scripted voice-mail message inviting patients to return her call. Otherwise, during the scripted telephone call, the study nurse reviewed the purpose and design of the study as described on the initial mailed invitation and invited the patient to participate if they passed initial eligibility screening (that is, had not previously undergone colorectal cancer screening). Interested and potentially eligible patients made an appointment to meet face-to-face with the study nurse for the eligibility screening. During the face-to-face visit, the study nurse reviewed detailed SCOPE trial eligibility criteria with the patient and answered questions from potential study participants about the study. Eligible, consenting patients were enrolled into the SCOPE trial.

Patients randomized to mail follow-up were mailed a second invitation inviting interested patients to call the SCOPE trial office to book an appointment for an identical eligibility screening visit with the study nurse. The mail follow-up invitation included the same content as the scripted telephone follow-up call.

### Outcome measures

The primary outcome was the attendance for eligibility screening with the study nurse. The secondary outcome was enrollment into the SCOPE trial.

### Sample size justification and statistical analysis

We calculated that a sample size of 1,360 individuals (680 in each group) would be needed to have 0.80 power at a 0.05 significance level to detect a 50% relative increase in the proportion of patients who attend for eligibility screening with the study nurse based on an assumed control event (attendance) rate of 10%. The recruitment substudy was stopped early, however, because of lack of ongoing funding for, and premature termination of the main SCOPE trial.

We compared the proportion of patients attending eligibility screening with the study nurse and the proportion of patients enrolled into the SCOPE trial in the telephone and mail follow-up groups, expressing our results as a relative risk and 95% confidence interval. The analysis was conducted according to the originally assigned groups, that is, all patients were included in the analysis regardless of whether they were successfully contacted (particularly relevant for the telephone arm) or not. A P-value of 0.05 was considered statistically significant.

This study received full approval from the Hamilton Health Sciences/McMaster Faculty of Health Sciences Research Ethics Board (reference # 09–147) and is in compliance with the Helsinki Declaration. The Research Ethics Board did not require informed consent for the comparison of mail and telephone recruitment strategies. Participants gave informed consent prior to their allocation to one of the three colorectal cancer screening strategies being compared (virtual colonoscopy, optical colonoscopy, or fecal occult blood testing).

## Results

**Figure 1:**
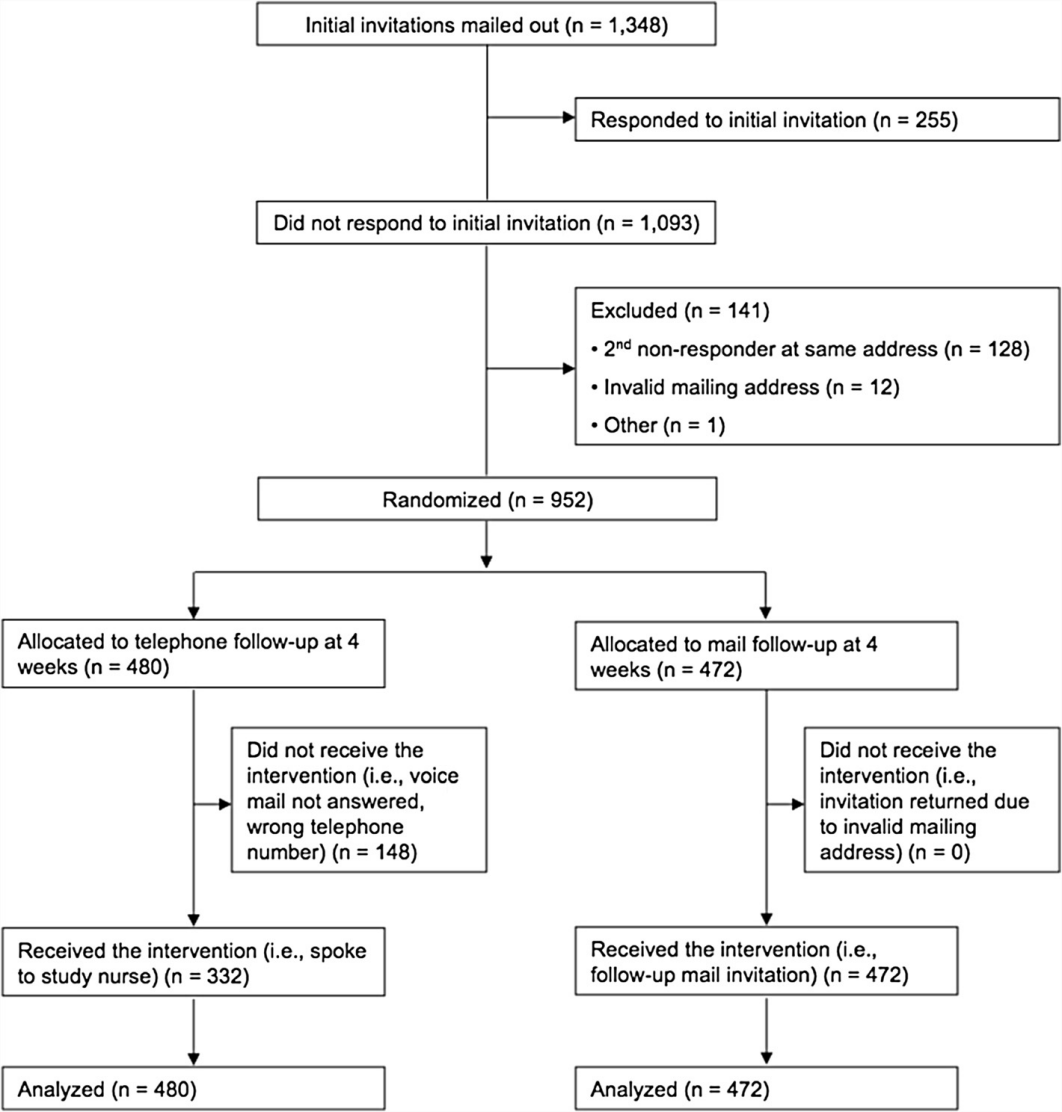
**Study flow diagram**.

From March to August 2010, we mailed letters to 1,348 patients from the five participating primary care practices inviting them to participate in the SCOPE trial. Of 255 patients who responded to the initial invitation, 104 patients were initially enrolled. At four weeks after the initial mailing, 952 eligible non-responders were randomized to a follow-up invitation by either telephone (N = 480) or mail (N = 472) (Figure 1). This recruitment substudy was stopped early because of lack of ongoing funding for the SCOPE trial. Participants in each group were similar in age (telephone group: mean age 55.7 years, standard deviation 6.1; mail group: mean age 55.6 years, standard deviation 6.3). 

The study nurse made contact (that is a live over-the-phone conversation) with 332 (69%) of the individuals randomized to telephone follow-up, of whom 60 (18.1%) were ineligible for the SCOPE trial due to recent colorectal screening (18 recent fecal occult blood testing, 39 recent optical colonoscopy, 1 recent virtual colonoscopy, 2 not specified). Of the remaining 272 individuals, 188 did not attend for eligibility screening with the study nurse either because they declined any further participation in the study or did not keep their eligibility screening appointment after initially booking one. A response was received from 178 (38%) of the individuals randomized to mail follow-up, of whom 37 (20.8%) were ineligible for the SCOPE trial due to recent colorectal cancer screening (14 recent fecal occult blood testing, 13 recent optical colonoscopy, 2 recent virtual colonoscopy, 8 not specified). Of the remaining 141 individuals, 98 did not attend for eligibility screening with the study nurse either because they declined any further participation in the study or did not keep their eligibility screening appointment after initially booking one. There were no crossovers between the telephone and mail follow-up groups.

Telephone follow-up resulted in a significantly greater proportion of patients attending eligibility screening with the study nurse and a greater proportion of patients enrolled into the SCOPE trial compared to mail follow-up (Table 1). Expressed as a ‘number needed to call’, our results demonstrate that for every 20 initial non-responders contacted by telephone, one additional participant was recruited to the clinical trial. 

**Table 1:** Outcomes for patients randomized to telephone or mail follow-up

	**Telephone (N = 480)**	**Mail (N = 472)**	**RR (95% Cl)**	***P- *value**
Attended for eligibility screening	84 (17.5)	43 (9.1)	1.92 (1.36, 2.71)	< 0.001
Enrolled into SCOPE trial	59 (12.3)	35 (7.4)	1.66 (1.11, 2.47)	0.01

Data reported as n (%), unless otherwise specified. RR, relative risk. 95% CI, 95% confidence interval.

## Discussion

Our study shows that patients who did not respond to our original mailed invitation were significantly more likely to attend for eligibility screening and to enroll in the SCOPE trial if contacted by a follow-up telephone call than by a mail reminder at four weeks after the initial invitation. Our findings are consistent with other randomized trials that have compared telephone-based reminders to alternative methods (no reminder or mail reminder) and found that telephone-based methods can increase recruitment by nearly two-fold [7,8]. Our study extends the evidentiary base in this field since it is, to our knowledge, only the second published randomized controlled trial comparing telephone to mail reminders in attempts to increase recruitment to clinical trials.

Investigators often face challenges when recruiting participants into clinical trials, particularly when the trial is evaluating a screening intervention which is directed at participants who do not have overt, symptomatic disease. We enrolled 66% more patients (in relative terms), or 4.9% more patients in absolute terms, using telephone follow-up with initial non-responders than by mail follow-up, translating to a ‘number needed to call’ of approximately 20. Because telephone follow-up is likely to be more resource intensive than mail-based follow-up, investigators will need to weigh the costs and benefits of a telephone-based recruitment strategy.

This study has some limitations. Firstly, the patients eligible for this study were non-responders to the initial mail invitations. This may potentially skew the results of the mail follow-up group as these patients have already been shown to be non-responsive to the initial mail invitations. Therefore, it is possible that an initial telephone-based invitation, as opposed to an initial mail-based invitation, could achieve greater results than what we observed in this study. Secondly, we did not measure the costs of each follow-up strategy in our study and did not systematically collect data on the percentage of patients who agreed to undergo the eligibility screening after each round of telephone calls, which would have helped us to estimate the cost and time for the telephone follow-up group. As such, we are unable to conduct a formal cost-benefit analysis. However, our results indicate that, as long as telephone follow-up can be conducted at less than 66% greater cost per invitation than mail follow-up (in relative terms), the cost per patient recruited would be lower with telephone follow-up. Thirdly, the telephone follow-up group received up to three attempts for contact compared to only one attempt in the mail follow-up group. Although from a logistic point of view, it made sense to call more than once before abandoning efforts to make contact with a potential participant, the different number of attempts in each group may have contributed to the lower response rate in the mail follow-up group. Thus, we make no comment about the relative effectiveness of making a single telephone call versus a single mail reminder to increase recruitment into a randomized controlled trial. Finally, the sample size of this substudy was restricted to only 952 subjects due to the premature termination of the larger SCOPE trial. However, this substudy of recruitment strategies (mail versus telephone reminders) was not stopped early based on the findings (that is it was not stopped early for benefit of either mail or telephone reminders). Despite the smaller sample size, the effect size we observed was sufficiently large (relative risk of 1.92) to be statistically significant and the only impact of our reduced sample size was greater imprecision around our point estimate of effect for the primary outcome (that is, wider 95% CI than we would have otherwise obtained with a larger study sample).

## Conclusions

We found that telephone-based follow-up led to a greater recruitment rate than mail-based follow-up of individuals who did not respond to an initial mailed invitation to take part in an RCT of colorectal cancer screening. Our findings should be of interest to investigators designing and conducting RCTs, particularly for screening interventions directed at otherwise healthy, asymptomatic individuals.

## Abbreviations

CI: confidence interval; PLCO: Prostate, Lung, Colorectal and Ovarian cancer screening trial; RCT: randomized control trial; RR: relative risk; SCOPE trial: Screening for Colorectal Cancer: a randomized trial of virtual colonoscopy, optical colonoscopy and fecal occult blood testing.

## Competing interests

All authors declare that they have no competing interests.

## Authors’ contributions

AW and JY had access to all the study data and take responsibility for the accuracy of the analysis. All authors had authority over manuscript preparation and the decision to submit the manuscript for publication. All authors read and approved the final manuscript.
